# Standardized mortality ratio in adults with congenital heart disease

**DOI:** 10.1093/ehjopen/oeaf165

**Published:** 2025-12-19

**Authors:** Elisabeth Hahlin, Christina Christersson, Peder Sörensson, Aleksandra Trzebiatowska-Krzynska, Zacharias Mandalenakis, Joanna Hlebowicz, Camilla Sandberg, Bengt Johansson, Daniel Rinnström

**Affiliations:** Department of Diagnostics and Intervention, Umeå University, 901 85 Umeå, Sweden; Department of Medical Sciences, Cardiology, Uppsala University, 75105 Uppsala, Sweden; Department of Medicine, Solna, Karolinska Institutet, 17177 Stockholm, Sweden; Departments of Cardiology and Medicine and Health Sciences, Linköping University, 58183 Linköping, Sweden; Department of Molecular and Clinical Medicine, Institute of Medicine, Sahlgrenska Academy, University of Gothenburg, 40530 Gothenburg, Sweden; Department of Cardiology, Clinical Sciences, Skåne University Hospital, 22100 Lund, Sweden; Department of Community Health and Rehabilitation, Umeå University, 901 85 Umeå, Sweden; Department of Diagnostics and Intervention, Umeå University, 901 85 Umeå, Sweden; Department of Diagnostics and Intervention, Umeå University, 901 85 Umeå, Sweden

**Keywords:** Mortality, Age-specific crude mortality rate, Age and gender standardized mortality rate

## Abstract

**Aims:**

The prevalence of adults with congenital heart disease (ACHD) is rising due to improved paediatric care. In parallel, updated data on prognosis in adult life are needed.

**Objectives:**

The aim was to calculate the standardized mortality ratio (SMR) and death rates in ACHD compared to the general population.

**Methods and results:**

Data were obtained from the national register of congenital heart disease. The general Swedish population served as a reference. SMR was calculated as the ratio between observed and expected deaths. 9089 patients (median age 28 years, interquartile range [IQR] 20–45, 47% females) were followed for a median of 8 years (IQR 4–14). 525 deaths occurred during observation. The SMR increased by lesion complexity: atrial septal defect [1.3 (95% CI: 1.1–1.5)]; ventricular septal defect [2.0 (1.4–2.7)]; congenital aortic valve disease [2.2 (1.6–2,9)]; Ebstein’s anomaly [3.2 (1.8–5.2)]; tetralogy of Fallot [3.8 (2.6–5.2)]; congenitally corrected transposition of the great arteries [5.6 (2.9–9.6)]; Eisenmenger syndrome [8.7 (5.5–13.1)]; transposition of the great arteries with a previous atrial redirection operation [12.3 (6.8–20.1)]; and Fontan physiology [22.5 (12.5–37.0)]. Calculations were also performed by severity (mild, moderate, and severe) and age by six age groups. SMR was generally higher in younger age, and the difference in mortality from the general population was estimated to be lower for older age groups. The mortality distribution and death rate per 1000 person-years have also been calculated for each lesion.

**Conclusion:**

The mortality in ACHD remains increased compared to the general population and reflects the severity of the lesion. In higher ages, the observed mortality is more in line with the general population, probably because of survival of the least affected patients, and that few persons with severe lesions have reached advanced age.

## Graphical Abstract

**Graphical Abstract:**
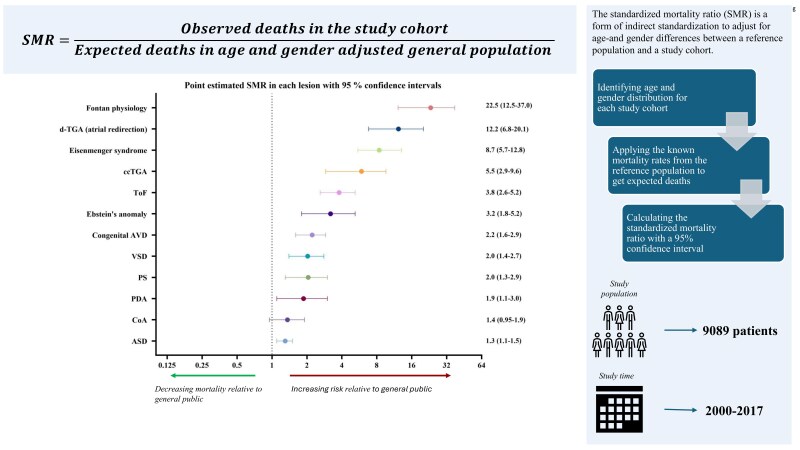

## Background

Advances in medical health care over the last half century have changed the survival rate, age profile, and comorbidity patterns for patients with congenital heart disease.^1–4^ More than 90% of all children with congenital heart defects (CHDs) are now reaching adulthood in industrialized countries, with a recent study reporting that 97% of patients survived until adulthood.^5^ An 88% rise in prevalence in adult congenital heart disease (ACHD) globally from 1990 to 2017 is described, with the greatest increase in prevalence in patients above 50 years of age.^4,6^ Two-thirds of the entire CHD population now consists of adults.^7^ This patient group has been estimated to continue to rise in prevalence for many decades to come.^8^ Consequently, the patient population will not only likely face cardiac comorbidities and complications that occur with older age,^9^ such as arrythmias,^1,10^ heart failure,^11^ infective endocarditis,^12–14^ pulmonary hypertension,^15,16^ and ischaemic stroke^17^ but also extracardiac complications such as dementia,^18^ cancer,^19,20^ kidney dysfunction,^21^ diabetes,^21,22^ and depression.^23^ This contributes to the disease burden that increases with age and very likely affects the prognosis. Without knowledge of the changing epidemiology and survival rates, the resources distributed to this medical field may not be sufficient to cover the needs of these patients, and patients may not be given adequate advice. Therefore, it is important to acquire data on current morbidity and mortality patterns.

In the present nationwide and register-based study, we have analysed the standardized mortality ratio (SMD), i.e. the ratio between observed and expected mortality, and death rates in ACHD. The results provide a contemporary image of the mortality patterns in this patient population.

## Methods

### Study design

This study was conducted as a register study covering the period 2000–2017. The aim was to calculate the SMD and death rate in patients with ACHD. The general population was used as a reference, and the major outcome during observation was death or heart transplant. The study was approved by the Swedish Ethical Review Authority (reference number 2020-00701).

### Data collection

All information regarding patients with ACHD was obtained from the Swedish national register on congenital heart disease (SWEDCON). All diagnoses in all patients were reviewed manually in relation to other available data.

**Figure 1:**
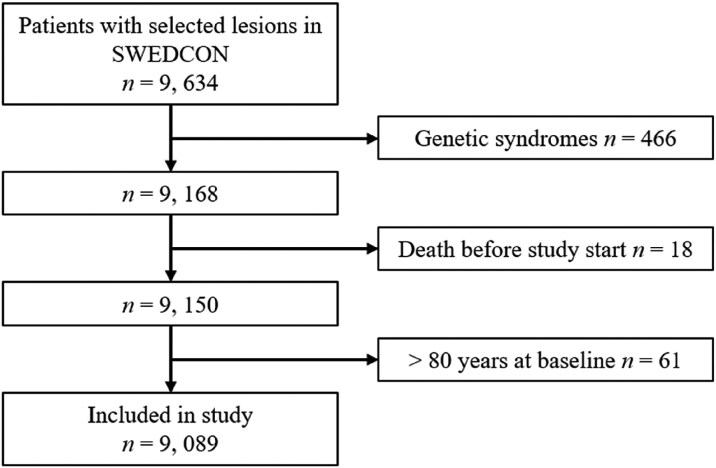
Inclusion process of patients in the order that they were included from the SWEDCON register. Selected lesions refer to Fontan physiology, *d-*Transposition of the great arteries, Eisenmenger syndrome, congenitally corrected transposition of the great arteries, tetralogy of Fallot, congenital aortic valve disease, Ebstein anomaly, ventricular septal defect, coarctation of the aorta, pulmonary stenosis, persistent ductus arteriosus, and atrial septal defect.

Inclusion criteria: 18–80 years of age, at least one clinic visit at an ACHD service and one of the following lesions: Fontan physiology, *d*-transposition of the great arteries (TGAs) with atrial redirection, Eisenmenger syndrome, congenitally corrected TGAs, tetralogy of Fallot (ToF), Ebstein’s anomaly, congenital aortic valve disease, ventricular septal defect (VSD), pulmonary stenosis, coarctation of the aorta, persistent ductus arteriosus (PDA), or atrial septal defect (ASD) (excluding PFO closure after an embolic stroke). Patients with genetic syndromes, *e.g.* Marfan, Turner or Down syndromes were excluded. Baseline was defined as the first clinic visit or, if the first visit occurred before, 1 January 2000. Patients were censored the year they turned 80 or at study end 31 December 2017. In *Figure 1*, the inclusion process is shown. For data on age and sex stratified mortality rates, population number, and ‘age at death’ in the general Swedish population, we used the human mortality database, where death rates stratified for sex and age in the general Swedish population are available with 1-year resolution.^24^

When classifying lesions into the subgroups mild, moderate, or severe, the ESC classification was applied.^25^ Patients with Eisenmenger syndrome, regardless of underlying heart lesion, were analysed as a separate category in the severe group. In cases with multiple diagnoses, the diagnosis with the highest severity was regarded as the main diagnosis and used in the analyses.

### Standardized mortality ratio

The calculation of the standardized mortality ratio (SMR) was performed according to the formula SMR = *O*/*E*, where *O* is the number of observed deaths and *E* is the number of expected deaths, if the general population had the same age and sex distribution as in the study cohort. The expected deaths will represent the sum of all individuals matched cumulative death rates over the study period.^25^ Standardization was performed by sex and age groups of 5 years, e.g. women 20–24 years of age in the age span 20–79 years of age, 18–19 being the only age group consisting of 2 years. A 95% confidence interval was calculated for each SMR value.

#### Interpretation

If the SMR is 2, twice as many deaths occurred in the study cohort compared with persons in the general population with the same distribution of age and sex, observed over a similar period. In contrast, an SMR below 1 indicates lower mortality than in the general population.

Observed and expected death rates were calculated by dividing the expected and observed death rates by the total patient years during observation. Since the total number of deaths in the general population and the total number of person-years in the general population are available at the *human mortality database*, the death rate by each age with 1-year increment can be calculated. These were used to find at which age the general population had a similar death rate as each severity group of lesions in six different age groups.

### Distribution of ‘age-at-death’

To visualize the distribution of deaths by age, all observed deaths in each lesion were divided into six age groups and then plotted as bar charts. To allow comparison with the general population, where 664 908 deaths occurred in the age span 18–79 years between 2000 and 2017, a random sample from these deaths was drawn. To find the minimum required 95% confidence and 5% marginal error sample size for those deaths, the central limit theorem was assumed and calculated by Cochran’s formula, with correction for a finite population, to *n* = 384. Simple random sampling was then performed in R. ‘Age-at-death’ for the 384 observed deaths was then plotted as a bar chart.

### Statistics

Hypothesis testing was done using the Poisson distribution, which will give the probability of a certain number of events occurring in a period when the expected count is known. The null hypothesis was rejected on *P* values < 0.05. To calculate 95% confidence intervals, the Garwood method was used, which uses the relationship between a Poisson parameter and the chi-square distribution.^26^ The significance level of the main study outcome SMR was Benjamin–Hochberg corrected to account for multiple testing with the false discovery rate set to 0.05. The confidence intervals were calculated according to the adjusted significance level (e.g. α = 0.0014 in Eisenmenger syndrome since it was the lowest-ranked *P* value). In total, 35 calculations of SMR were performed. All *P* values are two-tailed.

To test if the proportion of deaths that occurred before or after 50 years, an age where the death rates start to increase in the general population, the population was dichotomized at 50, and the Fisher’s exact test was applied.

The data from the SWEDCON register were handled in SPSS (version 28.0.1.1 (15)), but statistical analysis was performed in Microsoft Excel (Microsoft 365, version 2406, 64 bytes), except for Fisher’s exact test, which was performed in SPSS.

## Results

**Figure 2:**
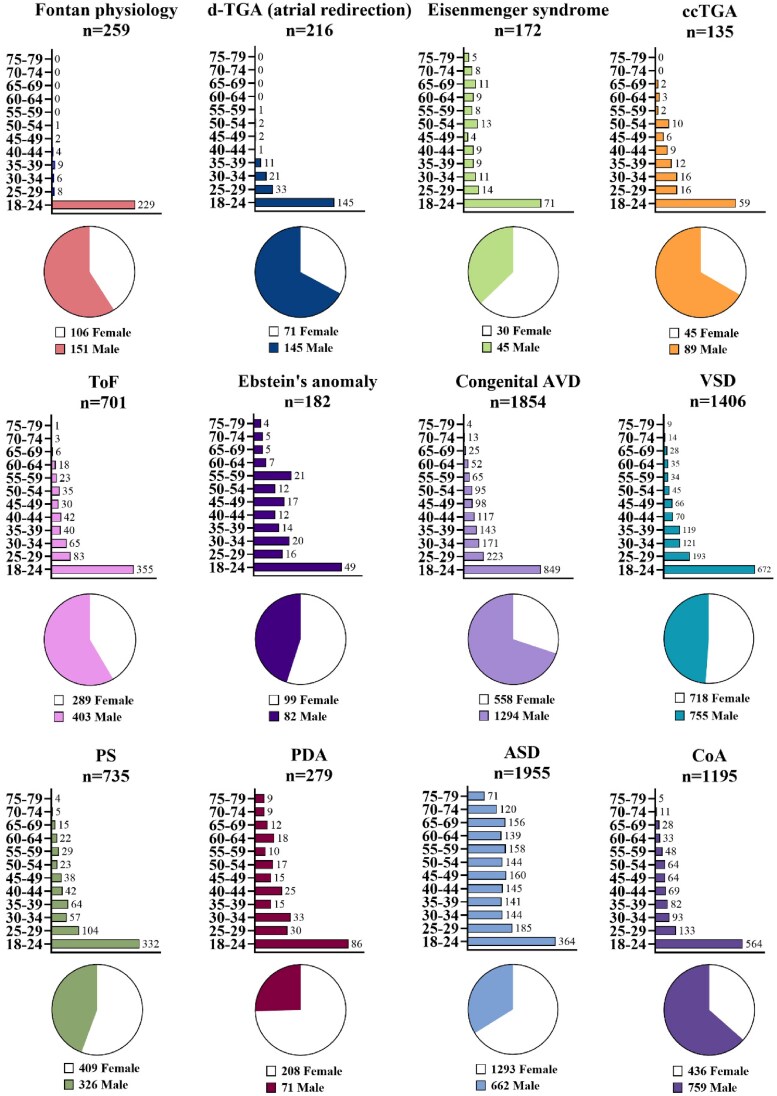
Age and sex of selected patients. Baseline refers to age at first visit or age at study start on 1 January 2000 if the first clinic visit was before 1 January 2000. The pie charts represent the percental distribution of sex in each lesion. *d-*TGA with atrial redirection, *d-*Transposition of the great arteries with atrial redirection; ccTGA, congenital corrected transposition of the great arteries; ToF, tetralogy of Fallot; congenital AVD, congenitally aortic valve disease; VSD, ventricular septal defect; PS, pulmonary stenosis; PDA, persistent ductus arteriosus; CoA, coarctation of the aorta; ASD, atrial septal defect.

In total, 9089 patients with ACHD (median age 28 years, interquartile range [IQR] 20–45, 47% females) were followed for a median of 8 years (IQR 4–14). 525 deaths occurred during observation in the register (*Figure 2*). The number of patients with each lesion and sex distribution is shown in *Figure 2*.

**Figure 3:**
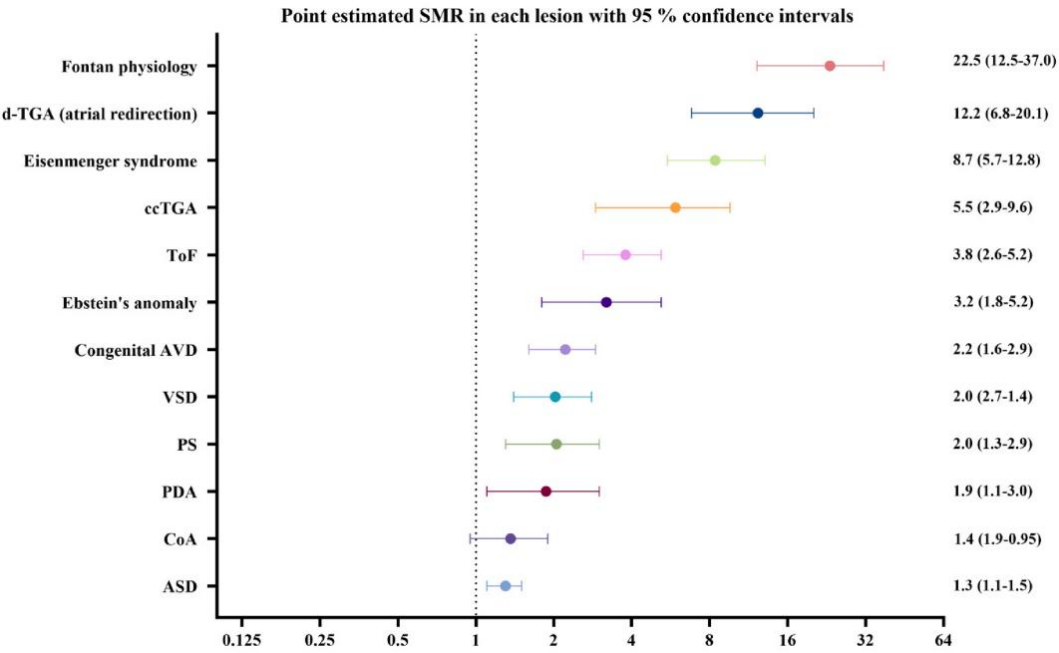
Point estimates for SMR with 95% confidence intervals in each lesion compared to the general population. Note that the *x*-axis is in a log-2 scale. The dotted vertical line at 1 represents an SMR of 1, meaning that the observed deaths were similar to the expected in an age- and sex-matched general population. A higher SMR means an increased death rate in patients than in the general population, while a lower SMR means a decreased death rate. *d-*TGA with atrial redirection, *d-*Transposition of the great arteries with atrial redirection; ccTGA, congenitally corrected transposition of the great arteries; ToF, tetralogy of Fallot; congenital AVD, congenital aortic valve disease; VSD, ventricular septal defect; PS, pulmonary stenosis; PDA, persistent ductus arteriosus; CoA, coarctation of the aorta; ASD, atrial septal defect.

**Figure 4:**
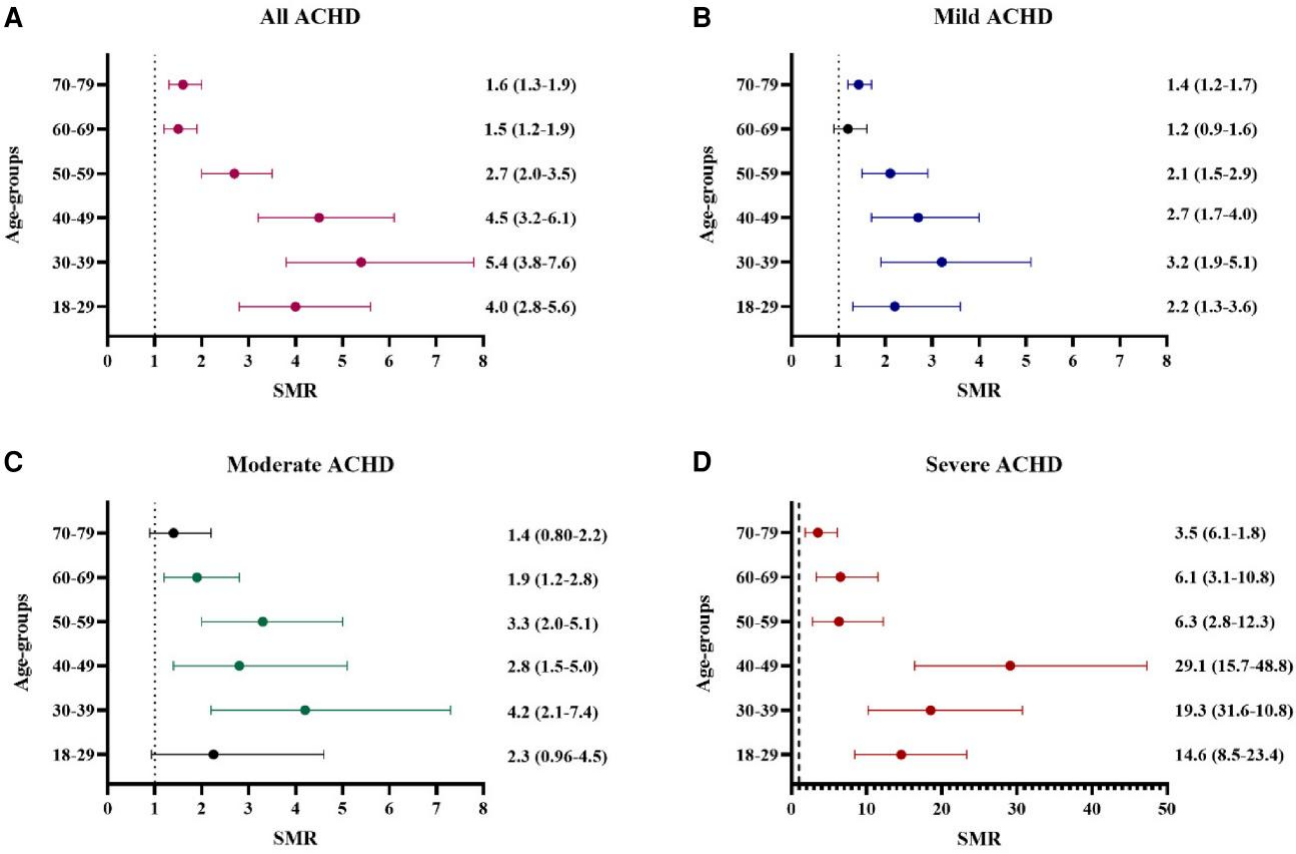
Point estimates for SMR in 6 age groups with 95% confidence intervals. Graph *a* represents all patients in the study, graph *b* the patients with mild defects, graph *c* the patients with moderate defects, and graph *d* the patients with severe defects. In our study, the defects congenital aortic valve disease, atrial septal defects, ventricular septal defects, pulmonary stenosis, and persistent ductus arteriosus were classified as mild defects. Coarctation of the aorta, Tetralogy of Fallot, and Ebstein’s anomaly were classified as moderate defects. Fontan physiology, *d-*transposition of the great arteries with atrial redirection, congenitally corrected transposition, and Eisenmenger syndrome were classified as severe. It is important to note the different scaling on the graphs and the fact that those in the severe group surviving after 60 years are primarily patients with Eisenmenger’s syndrome and congenitally corrected transposition of the great arteries.

**Figure 5:**
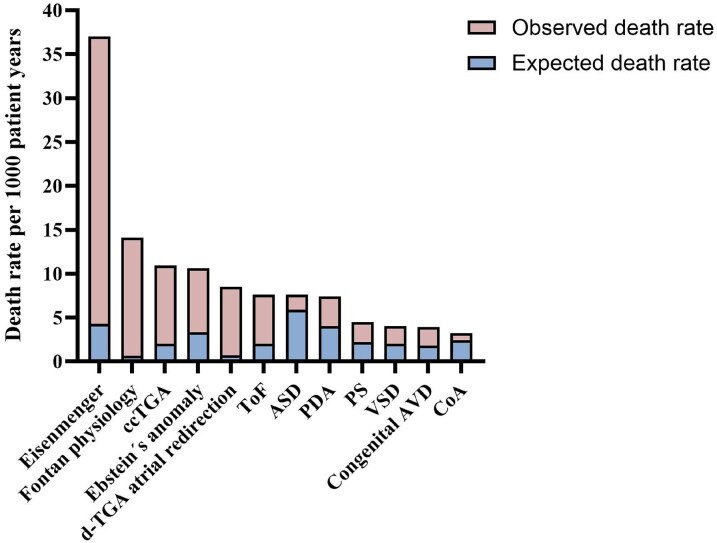
Deaths in each lesion per 1000 patient years. ccTGA, congenitally corrected transposition of the great arteries; *d-*TGA with atrial redirection, *d-*transposition of the great arteries with atrial redirection; ToF, tetralogy of Fallot; ASD, atrial septal defect; PDA, persistent ductus arteriosus; PS, pulmonary stenosis; VSD, ventricular septal defect; congenital AVD, congenital aortic valve disease; CoA, coarctation of the aorta.

All lesions, except coarctation of the aorta, have an increased SMR. The estimated SMR increases with the complexity of the heart lesion, with the lowest SMR in ASDs [1.3 (1.1–1.5)] and the highest in Fontan physiology [22.5 (12.5–37.0)] (*Figure 3*). The number of observed and expected deaths, i.e. the basis for SMR, are reported in *Table 1*. The subdivision of SMR in different age groups, which was done to account for the large age gap of 18–80 in the study, shows higher point estimates for SMR in younger ages that become less with increasing age (*Figure 4*). Eisenmenger syndrome had the highest crude death rate (*Figure 5*), but the order of lesions from highest to lowest crude death rate differed from that of the age and gender standardized SMR. Here, ASDs and ToF had a similar crude death rate (death rate per 1000 patient years), but the age at death was higher for ASDs (*Figure 5*).

**Table 1:** Standardized mortality ratio

CONGENITAL HEART LESION	EXPECTED DEATHS (*N*)	OBSERVED DEATHS (*N*)	SMR	*P* VALUE	BH CORRECTED SIGNIFICANCE LEVEL	95% CI BH CORRECTED
FONTAN PHYSIOLOGY	1.3	30	22.5	<0.001*	0.0042	12.5–37.0
*D*-TGA (ATRIAL REDIRECTION)	1.9	24	12.2	<0.001*	0.011	6.8–20.1
EISENMENGER SYNDROME	6.4	56	8.7	<0.001*	0.0014	5.7–12.8
CCTGA	3.1	17	5.5	<0.001*	0.021	2.9–9.6
TOF	14.6	55	3.8	<0.001*	0.014	2.6–5.2
EBSTEIN ANOMALY	5.9	19	3.2	<0.001*	0.032	1.8–5.2
CONGENITAL AVD	28.6	63	2.2	<0.001*	0.019	1.6–2.9
VSD	24.3	49	2.0	<0.001*	0.030	1.4–2.7
PS	14.8	30	2.0	<0.001*	0.038	1.3–3.0
PDA	9.6	18	1.9	0.02*	0.044	1.1–3.0
COA	26.5	36	1.4	0.091	0.047	0.94–1.9
ASD	99.3	128	1.3	0.0064*	0.043	1.1–1.5

All ACHD	EXPECTED DEATHS (*N*)	OBSERVED DEATHS (*N*)	SMR	*P* Value	BH CORRECTED SIGNIFICANCE LEVEL	95% CI BH CORRECTED
18–29	13.0	53	4.0	<0.001*	0.0125	2.8–5.6
30–39	11.4	63	4.5	<0.001*	0.0069	3.0–6.5
40–49	17.3	78	3.9	<0.001*	0.0056	2.7–5.4
50–59	31.8	87	2.7	<0.001*	0.015	2.0–3.5
60–69	63.4	100	1.6	0.0019*	0.035	1.2–1.8
70–79	91,4	144	1.6	<0.001*	0.022	1.3–1.9

MILD ACHD	EXPECTED DEATHS (*N*)	OBSERVED DEATHS (*N*)	SMR	*P*-VALUE	BH CORRECTED SIGNIFICANCE LEVEL	95% CI BH CORRECTED
18–29	7.7	16	2.2	0.0054*	0.042	1.3–3.6
30–39	7.1	23	3.2	<0.001*	0.023	1.9–5.1
40–49	11.6	31	2.7	<0.001*	0.025	1.7–4.0
50–59	22.4	47	2.1	<0.001*	0.026	1.5–2.9
60–69	47.7	57	1.2	0.21	0.049	0.9–1.6
70–79	79,0	113	1.2	<0.001*	0.036	1.2–1.7

MODERATE ACHD	EXPECTED DEATHS (*N*)	OBSERVED DEATHS (*N*)	SMR	*P* VALUE	BH CORRECTED SIGNIFICANCE LEVEL	95% CI BH CORRECTED
18–29	3.5	8	22.5	0.057	0.046	0.96–4.5
30–39	3.4	14	4.2	<0.001*	0.028	2.1–7.4
40–49	4.5	13	2.8	0.0018*	0.039	1.5–5.0
50–59	9.0	30	3.3	<0.001*	0.017	2.0–5.0
60–69	14.9	28	1.8	0.0031*	0.040	1.2–2.8
70–79	12.4	17	1.4	0.25	0.05	0.80–2.2

SEVERE ACHD	EXPECTED DEATHS (*N*)	OBSERVED DEATHS (*N*)	SMR	*P*-VALUE	BH CORRECTED SIGNIFICANCE LEVEL	95% CI BH CORRECTED
18–29	1.3	28	14.6	<0.001*	0.0097	8.45–23.4
30–39	1.9	26	18.5	<0.001*	0.0083	10.2–30.7
40–49	1,2	34	29.1	<0.001*	0.0028	16.4–47.4
50–59	0.9	10	4.2	<0.001*	0.029	2.8–12.3
60–69	0.64	15	6.5	<0.001*	0.018	3.3–11.5
70–79	4,0	14	3.5	<0.001*	0.033	1.8–6.1

SMR in each lesion and by severity with two-sided *P*-values, observed deaths in lesions, expected deaths, Benjamin–Hochberg corrected significance, and 95% confidence intervals. False discovery rate was set to 0.05.

*d-*TGA, *d-*transposition of the great arteries with atrial redirection; ccTGA, congenitally corrected transposition of the great arteries; congenital AVD, congenital aortic valve disease; VSD, ventricular septal defect; PS, pulmonary stenosis; PDA, persistent ductus arteriosus; CoA, coarctation of the aorta; ASD, atrial septal defect.

^*^Denotes significance.

For each category of severity of lesions, the corresponding age at which the general population had a similar death rate was calculated. For severe heart lesions, this comparable age was severely increased across all age groups, but calculations were not possible for patients aged over 70 years of age, due to the few patients reaching that age. In milder lesions, the comparable age was slightly increased in younger age groups but is in line with the general population in older patients (*Table 2*).

**Table 2:** Age in the general population with a similar death rate as different lesions

	18–29	30–39	40–49	50–59	60–69	70–79
Mild						
Age in general population	**42** (**31–46)**	**48** (**43–51)**	**53** (**50–56)**	**62** (**59–64)**	**66** (**63–68)**	**77** (**75–79)**
Median age in cohort	25	34	44	54	64	73
Patient years	16,228	13,297	9,587	6,788	5,343	3,032
Moderate						
Age in general population	**43 (19–49)**	**52 (44–57)**	**55 (47–60)**	**68 (62–71)**	**71 (66–74)**	**77 (70–80)**
Median age in cohort	24	34	44	54	64	72
Patient years	6,399	4,462	3,186	2,187	1,503	479
Severe						
Age in general population	**62 (56–65)**	**66 (60–70)**	**75 (70–77)**	**73 (63–78)**	**78 (72–82)**	**84 (77–87)**
Median age in cohort	24	34	43	54	63	72
Patient years	3,870	2,280	1,273	445	365	173

Each cell represents the age at which the general population has the most similar death rate as each severity group in a specific age group. For example, patients with severe congenital heart disease aged 18–29 had a death rate that was similar to that of a 62-year-old from the general population. A 95% confidence interval was calculated from the observed deaths. Mild lesions include atrial septal defects, ventricular septal defects, persistent ductus arteriosus, congenital aortic valve disease, and pulmonary stenosis. Moderate lesions include tetralogy of Fallot, Ebstein’s anomaly, congenitally corrected transposition of the arteries, and coarctation of the aorta. Severe lesions include Fontan physiology, congenitally corrected transposition of the great arteries, and *d-*transposition of the great arteries with atrial redirection. It is important to note that almost all surviving patients above 50 years of age in the severe category are congenitally corrected transposition patients and Eisenmenger syndrome as well as a low amount of patient years.

**Figure 6:**
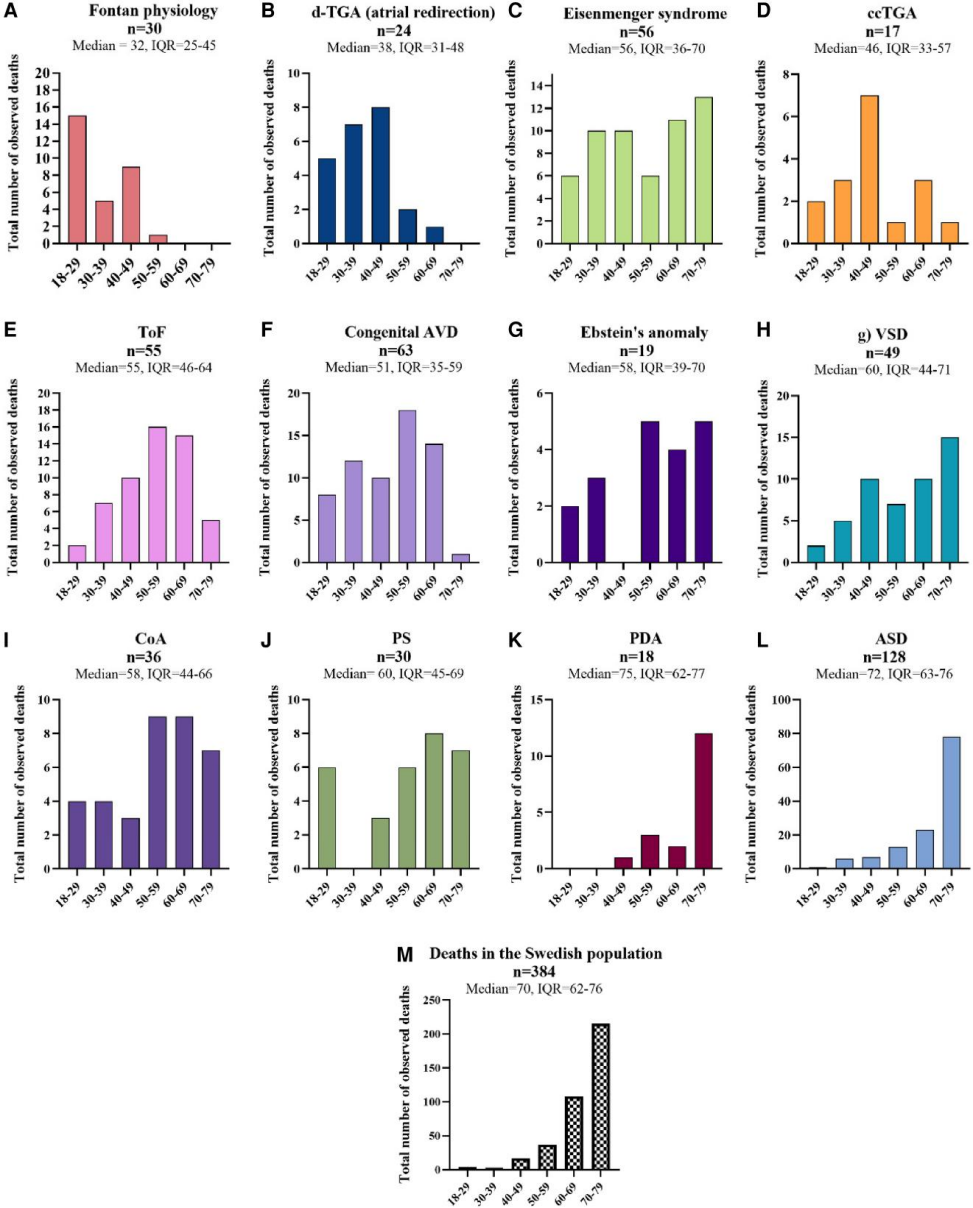
Distribution of ‘age at death’ by 6 age groups in a frequency bar chart. All 12 lesions and the Swedish general population are presented as an individual graph. The included deaths all occurred between the ages of 18–79. Severe lesions had an ‘age at death’ skewed towards younger ages compared to more simpler lesions such as PDA and ASD. The general population represents 384 randomly selected deaths from all that occurred in 18–79 years olds between 2000 and 2017. *d-*TGA with atrial redirection, *d-*Transposition of the great arteries with atrial redirection; ccTGA, congenital corrected transposition of the great arteries; ToF, tetralogy of Fallot; congenital AVD, congenital aortic valve disease; VSD, ventricular septal defect; CoA, coarctation of the aorta; PS, pulmonary stenosis; PDA, persistent ductus arteriosus; ASD, atrial septal defect.

In severe lesions, death occurred at a younger age, whereas in mild lesions, the age at death approximately followed the pattern in the general population. As the complexity of a lesion increased, the median and distribution of ‘age at death’ shifted towards younger ages (*Figure 6*). All lesions except atrial defects and PDA had a higher risk ratio of death occurring before the age of 50. The risk ratio generally followed the severity of the lesion and increased with the complexity of the lesion (*Table 3*).

**Table 3:** Death before and after the age of 50 years relative to the general population

Lesion	Total deaths (*n*)	Deaths before 50 (*n*)	Deaths after 50 (*n*)	*P* value	Corrected significance level	Risk ratio with CI
General population	384	24	360	—	—	—
Fontan physiology	30	29	1	<0.001	0.0042	15.3 (10.3–22.7)
*d-*TGA (atrial redirection)	24	21	3	<0.001	0.0083	13.9 (9.1–21.0)
Eisenmenger syndrome	56	26	30	<0.001	0.0125	7.4 (4.5–11.9)
ccTGA	17	12	5	<0.001	0.017	11.2 (6.8–18.3)
ToF	55	19	36	<0.001	0.021	5.5 (3.2–9.3)
Ebstein anomaly	19	5	14	0.008	0.025	5.3 (1.7–15.9)
Congenital AVD	63	30	33	<0.001	0.029	7.5 (4.7–12.0)
VSD	49	17	32	<0.001	0.033	5.5 (3.2–9.5)
CoA	36	11	25	<0.001	0.038	4.8 (2.6–9.0)
PS	30	9	21	<0.001	0.042	4.8 (2.4–9.3)
ASD	128	14	114	0.12	0.046	1.7 (0.9–3.2)
PDA	18	1	17	∼1	0.05	0.9 (0.1–6.8)

Age at death in lesions relative to the general population. An increased risk of death occurring before 50 in all lesions relative to the general population, except in PDA and ASD. *P* values and risk ratios were calculated using Fisher’s exact test. A 95% CI was constructed for each risk ratio. Holm corrected *P* values indicate the level of statistical significance after correction.

*d-*TGA, *d-*Transposition of the great arteries with atrial redirection; ccTGA, congenital corrected transposition of the great arteries; congenital AVD, congenital aortic valve disease; VSD, ventricular septal defect; PS, pulmonary stenosis; CoA, coarctation of the aorta; ASD, atrial septal defect; PDA, persistent ductus arteriosus.

## Discussion

Although survival into adulthood has improved during the last decades, mortality remains increased, especially in patients with severe heart lesions. In general, the excess mortality was more pronounced in younger patients. This difference decreased with age, suggesting a relatively good prognosis in patients with mild to moderate lesions, who have survived until middle age. In mild lesions, especially older patients seem to follow the mortality distribution of the general population.

SMR has many advantages when examining mortality in congenital heart disease, as it is a form of indirect standardization, allowing for calculations in small subgroups with skewed age distributions. Another major advantage is that no controls are needed. With reservations for borderline outcome in coarctation of the aorta, we found an increased mortality in all lesions, and SMR increased gradually with increased severity of the underlying congenital heart lesion. However, the point estimate for SMR regarding coarctation of the aorta is in line with other lesions with low SMR and should probably be interpreted in a corresponding way, i.e. slightly increased SMR but near the general population. Similar results have been reported, especially regarding moderate and severe lesions^27^ but in mild lesions, such as ASDs and VSDs, the previous report did not reveal an increased SMR.^27^ This difference may be explained by the larger population and more, twice as many, fatal outcomes included here in our study, enhancing the statistical power and thus the granularity.

In addition to SMR, we also calculated the death rate in different age groups and by severity, with a 95% confidence interval, estimating the age at which the general population had a similar death rate. Also here, the excess mortality fades in higher ages. In this context, it must be noted that the patients who survive into older ages may be less affected by the CHD and that the mortality in the general population also increases with age, both factors that may act in the direction to reduce the difference between the groups. Notably, in some groups of patients, no individuals have yet reached a higher age, as in patients with a previous Fontan operation, where the highest achieved age in the register between 2000 and 2017 was just below 60 years.

Here, we have also examined the age distribution at death in specific lesions relative to the general population. We can therefore show a risk ratio associated with death occurring before the age of 50 in each lesion compared to the general population. In all lesions except PDA and ASDs, the risk of death occurring before the age of 50 differed from that of the general population, with an increased risk of death occurring at younger ages.

### Limitations

This is a register study and thus limited to patients within the register. However, the number of patients, follow-up time, and multi-centre mode compensate for this limitation in many aspects. Sweden is a high-income country with public financing of health care, and therefore, the results may not be generally applicable.

Although the study includes all specialized centres and hospitals in Sweden, the number of patients with severe congenital heart lesions is still relatively small and therefore creates large confidence intervals when age-stratified.

Though we lack data on the cause of death, all-cause mortality is generally considered a relevant outcome measure, taking into account the complete spectrum of diseases and unnatural death. In older patients, ‘survivor effects’ may attenuate relative risks when compared to the general population.

Both Fisher’s exact test and Garwood’s confidence interval for a Poisson parameter are conservative methods, which minimize the risk of falsely rejecting the null hypothesis but also somewhat increase the risk of type II errors. The Benjamin–Hochberg procedure is a method that controls type 1 errors but does not unlike the Holm and Bonferroni procedure inflate type II errors. Despite this, all but one of the SMRs had a confidence interval separated from 1.

## Conclusions

The mortality in congenital heart disease remains increased and in some lesions exceeds a 20-fold increase, and in general reflects the severity of the congenital heart lesion. In some mild heart lesions, e.g. ASD and PDA, the age at death reflects the general population, with most deaths in higher ages, in contrast to severe lesions, where an important proportion of patients still die in early adulthood.

## Lead author biography


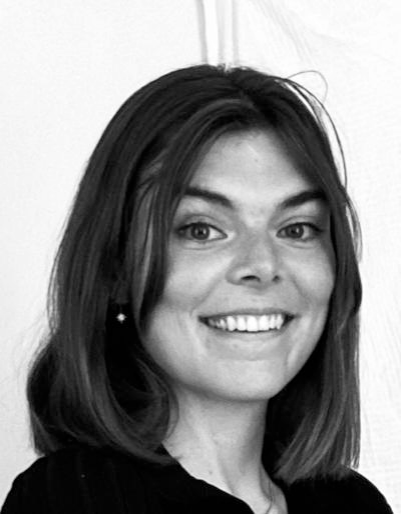


Elisabeth Hahlin completed her medical degree in 2024 and initiated her doctoral studies during the same year. In 2025, she commenced her medical internship, combining clinical training with ongoing academic research. Her doctoral thesis focuses on ACHD, with a particular emphasis on mortality outcomes.

## Data Availability

All data used in this study were obtained from the national health care register Swedcon. In addition to approval from the ethics review board, access to these data is governed by national regulations and data-use agreements. As a result, the datasets cannot be shared publicly or transferred to third parties. Data may, however, be assessed collaboratively upon reasonable request, whereby aggregated information can be reviewed together with the authors in a manner consistent with applicable regulations.
